# Marginal dietary zinc deprivation augments sepsis‐induced alterations in skeletal muscle TNF‐*α* but not protein synthesis

**DOI:** 10.14814/phy2.13017

**Published:** 2016-11-03

**Authors:** Kristen T. Crowell, Shannon L. Kelleher, David I. Soybel, Charles H. Lang

**Affiliations:** ^1^ Department of SurgeryCollege of MedicinePennsylvania State UniversityHersheyPennsylvania; ^2^ Department of Cellular and Molecular PhysiologyCollege of MedicinePennsylvania State UniversityHersheyPennsylvania; ^3^ Department of PharmacologyCollege of MedicinePennsylvania State UniversityHersheyPennsylvania; ^4^ Department of Nutritional SciencesPennsylvania State UniversityUniversity ParkPennsylvania

**Keywords:** Inflammation, muscle wasting, sepsis, skeletal muscle metabolism, zinc deficiency

## Abstract

Severe zinc deficiency is associated with an increased systemic inflammatory response and mortality after sepsis. However, the impact of mild zinc deficiency, which is more common in populations with chronic illnesses and sepsis, is unknown. In this study, we hypothesized that marginal dietary Zn deprivation (ZM) would amplify tissue inflammation and exacerbate the sepsis‐induced decrease in muscle protein synthesis. Adult male C57BL/6 mice were fed a zinc‐adequate (ZA) or ZM diet (30 or 10 mg Zn/kg, respectively) over 4 weeks, peritonitis was induced by cecal ligation and puncture (CLP), and mice were examined at either 24 h (acute) or 5 days (chronic) post‐CLP. Acute sepsis decreased the in vivo rate of skeletal muscle protein synthesis and the phosphorylation of the mTOR substrate 4E‐BP1. Acutely, sepsis increased TNF‐*α* and IL‐6 mRNA in muscle, and the increase in TNF‐*α* was significantly greater in ZM mice. However, muscle protein synthesis and 4E‐BP1 phosphorylation returned to baseline 5 days post‐CLP in both ZA and ZM mice. Protein degradation via markers of the ubiquitin proteasome pathway was increased in acute sepsis, yet only MuRF1 mRNA was increased in chronic sepsis and ZM amplified this elevation. Our data suggest that mild zinc deficiency increases TNF‐*α* in muscle acutely after sepsis but does not significantly modulate the rate of muscle protein synthesis.

## Introduction

Sepsis produces a catabolic state in skeletal muscle characterized by protein degradation in excess of protein synthesis. When sepsis‐induced inflammation is protracted, the imbalance leads to a net loss of skeletal muscle mass (Lang et al. 2007). The resulting erosion of muscle is associated with longer hospital stays, increased ICU time, and inability to wean from the ventilator (de Jonghe et al. 2009; Hill 1998; Callahan and Supinski 2009). The sepsis‐induced decrease in muscle protein synthesis is mediated at least in part by a reduction in both peptide‐chain initiation and translational efficiency (Vary and Kimball 1992). A major contributor to the sepsis‐induced decline in translation is the inhibition of the mammalian target of rapamycin (mTOR), a Ser/Thr kinase which integrates various hormone, nutrient, and energy signals. As a result of the decreased kinase activity, the phosphorylation of downstream substrates of mTOR, 4E‐binding protein 1 (4E‐BP1), and ribosomal protein S6 kinase 1 (S6K1), is subsequently decreased (Lang and Frost 2006, 2004; Lang et al. 2010). The sepsis‐induced elevation of the inflammatory cytokine tumor necrosis factor (TNF)‐*α* and glucocorticoids drives this reduction in mTOR activity, with other mediators having permissive effects (Lang and Frost 2006, 2005). Although the short‐term catabolic state can be viewed as advantageous to the host as it leads to an increased release of amino acids which supports hepatic protein synthesis and immune function, it also impedes recovery (Hasselgren et al. 1988; Sax et al. 1988).

Zinc (Zn) is a vital micronutrient required for a wide variety of cellular functions and is an integral part of the response to illness (Prasad 1967, 2013). Zn intake and excretion are highly regulated to maintain both whole‐body and cellular Zn homeostasis (Prasad 2013; King et al. 2000; Johnson et al. 1993). In the presence of inadequate Zn intake, excretion of this essential mineral decreases to maintain Zn balance (Wada et al. 1985; Wallwork and Sandstead 1983; Wallwork et al. 1983). However, once Zn intake falls below a critical threshold, compensation is inadequate and total body Zn stores are decreased (Johnson et al. 1988). Mild Zn deficiency (ZD) has a seemingly normal phenotype until stressors, such as abdominal surgery, critical illness, or reproduction (pregnancy and lactation), unmask signs of mild ZD such as impaired immune function, decreased lean body mass, and oligospermia (Phillips et al. 2015; McCormick et al. 2015; Dempsey et al. 2012; Bostanci et al. 2015). Compared to the overt symptoms produced by severe ZD, which include dermatitis, weight loss, alopecia, increased risk of infections, impaired wound healing, and hypogonadism (Prasad 2013; King et al. 2000; Johnson et al. 1993), the phenotype of mild ZD is more subtle. Severe ZD is now rare in developed countries, yet mild Zn imbalances still persist and may be more common in patients with underlying medical comorbidities, such as diabetes and chronic inflammation in rheumatoid arthritis (Al‐Timimi et al. 2014; Mierzecki et al. 2011; Milanino et al. 1993).

In murine models, animals fed a severely Zn‐deficient diet show symptoms and signs similar to severe ZD in humans (Wallwork and Sandstead 1983; Wallwork et al. 1983; Giugliano and Millward 1984). In response to sepsis, Zn is redistributed from the plasma to vital organs such as the liver in both human studies and experimental animal models (Gaetke et al. 1997; Pekarek and Beisel 1969; DiSilvestro and Cousins 1984). Prior work by Knoell et al. 2009 reported that severe ZD in mice, augments the sepsis‐induced decline in plasma Zn, and blunts the Zn accumulation in these organs after cecal ligation and puncture (CLP). Moreover, mortality after sepsis was higher in severe ZD mice, and the immune response was exacerbated as oxidative stress and cell death increased in the liver and lung, and plasma cytokines were elevated (Knoell et al. 2009). Additionally, disturbances in Zn homeostasis during sepsis may also impair mTOR signal transduction and protein synthesis (McClung et al. 2007; Lynch et al. 2001). In contrast, mild ZD has yet to be explored in the setting of sepsis or in vivo regarding sepsis‐induced changes in muscle protein synthesis. Therefore, this study was designed to test the hypothesis that mild Zn dietary depletion (ZM) will amplify the local inflammatory response within skeletal muscle following sepsis and thereby exacerbate the sepsis‐induced decrease in muscle protein synthesis.

## Methods

### Animal protocol

Viral antibody‐free male C57BL/6 mice (Taconic, Hudson, NY) aged 10–12 weeks (26.9 ± 2.0 g) were acclimated for 1 week prior to the experiment and allowed standard rodent chow (Teklad Globak 2019, Harlan Teklad, Boston, MA) and water ad libitum. Mice were individually housed in polycarbonate cages with corncob bedding and maintained in a controlled environment with a 12:12 h light–dark cycle. All experiments were approved by the Institutional Animal Care and Use Committee at the Pennsylvania State University College of Medicine and adhered to National Institutes of Health (NIH) guidelines. After acclimation, cages were changed, and purified diets (MP Biomedical, Santa Ana, CA) were provided containing either Zn‐adequate (30 mg/kg Zn; ZA) or marginally Zn‐deficient (10 mg/kg Zn; ZM) diets. The Zn concentration of each diet was verified by atomic absorption spectrophotometry. Diets were provided ad libitum for 4 weeks. We have previously shown that this model of dietary zinc deprivation can be used to induce ZM (Croxford et al. 2011). Food intake and animal weights did not differ during the 4 weeks of dietary Zn restriction compared to the respective ZA groups.

### Acute and chronic sepsis model

The CLP model of sepsis was chosen over endotoxin injections as alteration in muscle turnover persist multiple days following CLP (Breuille et al. 1998) yet are only transient following endotoxin, and resistance can develop after multiple doses or a sustained infusion of endotoxin (Jepson et al. 1986; Ash and Griffin 1989). Sepsis was induced by CLP on randomly assigned mice from each dietary group. Mice were anesthetized with isoflurane (2–3% in O_2_ with 1.5% maintenance; Vedco, St. Joseph, MO), the abdomen was shaved, cleaned with betadine, and a 1‐cm midline incision was made. The distal one‐third of the cecum was ligated with a 4‐0 silk suture, punctured twice with a 23 gauge needle, squeezed to extrude some feces, and returned into the peritoneal cavity. The abdominal wall and skin were closed separately, and animals were resuscitated with 1 ml subcutaneous injected sterile 0.9% saline. Nonseptic mice were subjected to the same protocol except the cecum was identified and returned into the peritoneal cavity without further manipulation.

After surgery, nonseptic and septic mice were provided free access to water. In the first experimental series (acute sepsis), the effect of acute sepsis was studied in mice 24 h post‐CLP (*n *= 40; 8 nonseptic mice/diet and 12 septic mice/diet). Food was withheld from all mice after surgery to account for minimal food intake following CLP in the septic mice. Survival was 92% (11 of 12 septic mice survived) at the completion at 24 h in both ZA and ZM mice, and 100% of nonseptic mice survived. In the second experimental series (chronic sepsis), a separate group of mice (*n *= 59; 9–10 nonseptic mice/diet; 20 septic mice/diet) were observed for 5 days post‐CLP or nonseptic laparotomy. In this experiment, the mice were continued on their respective ZA or ZM diet for all 5 days. At the completion of the 5 days, although survival after sepsis trended toward decreased survival in ZM mice, there was no statistical difference in survival between ZA and ZM (60 and 35%, respectively; *P *= 0.1).

### Sample collection

At the end of the 24 h or 5‐day study, surviving mice were anesthetized with isoflurane (as above) for specimen collection and then killed. The gastrocnemius and quadriceps were removed, weighed, and cooled to the temperature of liquid nitrogen. Blood was collected in a heparinized syringe from the inferior vena cava, centrifuged, and plasma was collected. Muscles and plasma were stored at −70°C. This study focused on changes in the gastrocnemius, a predominantly fast‐twitch muscle, although it is acknowledged that sepsis‐induced changes in predominantly slow‐twitch muscles (e.g., soleus) may differ (Muthny et al. 2008).

### In vivo protein synthesis

The rate of protein synthesis in gastrocnemius was determined in vivo by the flooding dose technique, as previously described (Vary and Kimball 1992; Vary and Lang 2008). Mice from both acute and chronic sepsis groups were anesthetized with isoflurane 12 min after intraperitoneal injection of [^3^H]‐L‐phenylalanine (Phe; 150 mmol/L, 30 *μ*Ci/mL; 1 mL/100 g body weight). A blood sample was collected at 15 min after injection of the radiolabeled tracer and used for determination of Phe concentrations and radioactivity. Blood samples were centrifuged (13 000 × *g* for 1 min at 4°C), and the plasma was collected for analysis. The gastrocnemius was rapidly excised after blood collection and frozen as above. The specific radioactivity of TCA deproteinized plasma was determined by HPLC (Vary and Lang 2008). Specific radioactivity was calculated by dividing the radioactivity of the Phe peak by the concentration of Phe in the sample. A portion of powdered muscle was homogenized in ice‐cold PCA, and the supernatant was used to estimate the rate of incorporation of [^3^H]Phe into protein. The specific radioactivity was calculated by dividing the amount of radioactivity in the peak corresponding to Phe by the concentration of the amino acid in the same fraction. Rates of protein synthesis were calculated using the plasma Phe‐specific activity as the precursor pool, and rates were expressed as nmol Phe/hour/mg tissue protein.

### Western blot analysis

The gastrocnemius was prepared as previously described (Lang and Frost 2006; Steiner et al. 2014). Briefly, a portion of the muscle was homogenized in an ice‐cold homogenizing buffer (20 mmol/L HEPES, pH 7.4, 2 mmol/L EGTA, 50 mmol/L NaF, 100 mmol/L KCl, 0.2 mmol/L EDTA, 50 mmol/L *β*‐glycerophosphate, 1 mmol/L DTT, 0.1 mmol/L PMSF, 1 mmol/L benzamidine, 0.5 mmol/L sodium vanadate). Protein content was quantified using Pierce BSA protein assay (Thermo Scientific, Rockford, IL). Equal concentrations of protein were subjected to electrophoresis on a 12.5% polyacrylamide gel, and proteins were transferred to PVDF membrane. Blots were incubated with primary antibodies from Cell Signaling Technology (Beverly, MA): total 4E‐BP1, total and phosphorylated (Thr389) p70 S6K1, protein light chain 3 (LC3)‐A/B, and *β*‐tubulin. Blots were then washed and incubated with secondary antibody (horseradish peroxidase‐conjugated goat anti‐rabbit IgG) and visualized with enhanced chemiluminescence (ECL) reagents (Pierce Chemical, Rockford, IL) according to manufacturer's instruction. Blots were imaged using FluorChem (ProteinSimple, San Jose, CA) and densities in the linear range were quantified using Image J (NIH, Bethesda, MD).

### RNA extraction and real‐time quantitative PCR

Total RNA was isolated using Tri‐reagent (Molecular Research Center, Cincinnati, OH) and RNeasy mini kit (Qiagen, Valencia, CA) according to manufacturers’ protocol, as previously reported (Lang et al. 2012). Briefly, gastrocnemius was homogenized in Tri‐reagent followed by chloroform extraction. An equivalent volume of 70% ethanol was added to the aqueous portion and loaded to the Qiagen mini‐spin column. The protocol of the Qiagen mini‐kit protocol was then performed including the on‐column DNase I treatment. RNA was eluted from the column with RNase‐free water and quantified (NanoDrop 2000, Thermo Fisher Scientific, Waltham, MA). RNA quality was evaluated on 1% agarose gel. Total RNA was reverse transcribed using superscript III reverse transcriptase (Invitrogen, Carlsbad, CA). Real‐time quantitative PCR was performed using 25 ng of cDNA in StepOnePlus system using TaqMan gene expression assay (Applied Biosystems, Foster, CA) for: atrogin‐1 (NM_026346.2), muscle RING‐finger 1 (MuRF1; NM_001039048.2), interleukin (IL)‐6 (Mm00446190_m1), TNF‐*α* (Mm00443258_m1), ribosomal protein L32 (rpL32; Mm02528467_g1), and DNA‐directed RNA polymerase II polypeptide A (Polr2a; Mm00839493_m1). The comparative quantitation method 2^−∆∆Ct^ was used to present gene expression normalized to the endogenous control, rpL32 for the acute sepsis study and Polr2a for chronic sepsis studies. In chronic sepsis, rpL32 was not used as a control due to a significant variation in the mRNA content between groups, but Polr2a did not differ between groups.

### Statistics

Data are presented as the mean ± standard error of mean (SEM), where the number of mice per group is presented in the figure or table legends. Statistical analysis of the data was performed using two‐way analysis of variance (ANOVA) followed by post hoc Student–Newman–Keuls (SNK) test when appropriate (GraphPad Prism version 6.0, La Jolla, CA). Differences between groups were considered significant when *P *< 0.05.

## Results

### Muscle weights

Sepsis did not alter body weight 24 h after CLP compared to fasted, time‐matched nonseptic mice fed the ZA diet (Table 1). The weight of the gastrocnemius and quadriceps did not differ between nonseptic and septic mice at the 24‐h time point. In contrast, 5 day after CLP, body weight and muscle weights of septic mice were decreased, compared to time‐matched ZA‐nonseptic mice. There was no effect of mild dietary zinc restriction on body or muscle weight in either septic or nonseptic mice (Table 1).

**Table 1:** Body weight and muscle weights after sepsis

	Nonseptic		Septic		Two‐way ANOVA		
	ZA	ZM	ZA	ZM	Interaction	Sepsis	Diet
Acute sepsis	(*n *= 8)	(*n *= 8)	(*n *= 11)	(*n *= 11)			
Body weight (g)	24.7 ± 0.7	24.1 ± 0.8	24.4 ± 0.7	24.4 ± 0.5	0.7	1	0.7
Gastroc (mg)	136.4 ± 3	131.1 ± 3	140.7 ± 3	139.1 ± 3	0.6	0.06	0.3
Quad (mg)	163 ± 5	164 ± 4	154 ± 4	158 ± 4	0.8	0.09	0.7
Chronic sepsis	(*n *= 9)	(*n *= 10)	(n = 12)	(*n *= 7)			
Body weight (g)	25.6 ± 0.5^a^	25.5 ± 0.5^a^	23.3 ± 0.5^b^	23.6 ± 0.8^b^	0.5	0.006	0.7
Gastroc (mg)	151.7 ± 3^a^	151.3 ± 4^a^	133.5 ± 3^b^	132.4 ± 3^b^	0.8	<0.0001	0.5
Quad (mg)	182.2 ± 6^a^	178.7 ± 5^a^	158.2 ± 4^b^	155.3 ± 5^b^	1	0.003	0.7

Body weights were measured after CLP or sham operation after an overnight fast in the acute sepsis study or after 5 days with food ad libitum in chronic study. Gastrocnemius (gastroc) and quadriceps (quad) were freeze clamped then weighed. Values are mean ± SE. Each category was analyzed using two‐way analysis of variance (ANOVA) and post hoc Student–Newman–Keuls test; values with different letters are significantly different (*P *< 0.05) in post hoc testing.

### Protein synthesis

**Figure 1:**
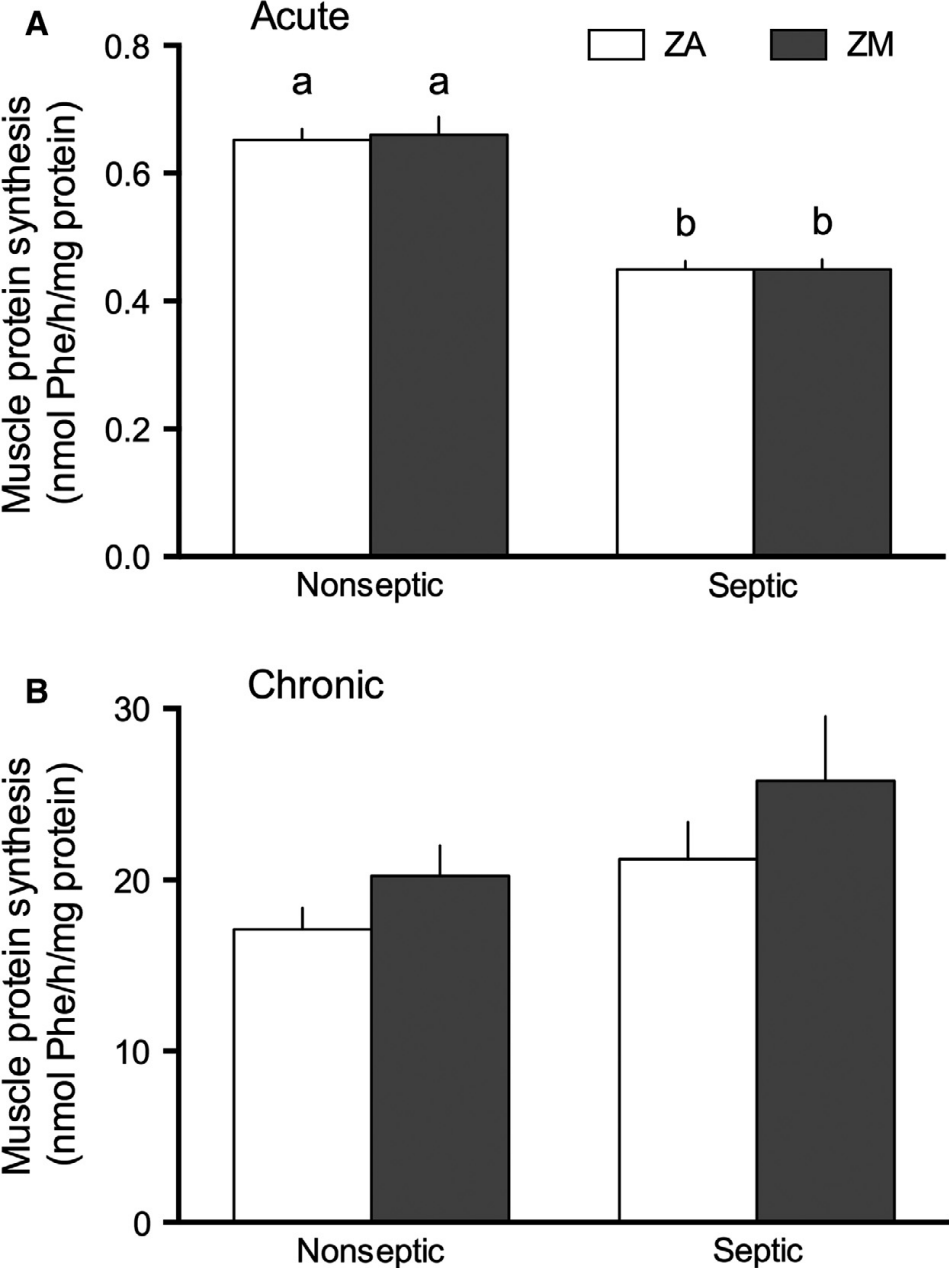
Effect of ZM and sepsis on skeletal muscle protein synthesis. In vivo protein synthesis was measured using the flooding dose technique with radiolabeled phenylalanine in gastrocnemius at 24 h (Acute sepsis; A) or 5 days (Chronic sepsis; B) after sham or septic laparotomy. The effect of acute and chronic sepsis (*P *< 0.001 and *P *= 0.046, respectively) was significant using a two‐way analysis of variance (ANOVA), while effects of diet or interaction were not significant. Values with different letters are significantly different, *P *< 0.05. Values are mean ± SEM;* n *= 7–14 mice/group.

Acute sepsis decreased global protein synthesis in the gastrocnemius 30% compared with time‐matched values from nonseptic mice (Fig. 1A). Contrary to our expectations, the acute sepsis‐induced decrease in muscle protein synthesis was not influenced by dietary Zn restriction. In chronic sepsis, a sepsis effect was seen via two‐way ANOVA; however, this trend toward an increase in the rate of muscle protein synthesis was not confirmed in post hoc testing as there were no differences between chronic septic and nonseptic groups, regardless of Zn content in the diet (Fig. 1B). Moreover, maintaining mice on the ZM diet for 4 weeks did not alter muscle protein synthesis at either the early (24 h; Fig. 1A) or late (5 days; Fig. 1B) time points following a nonseptic laparotomy.

### mTOR signaling

**Figure 2:**
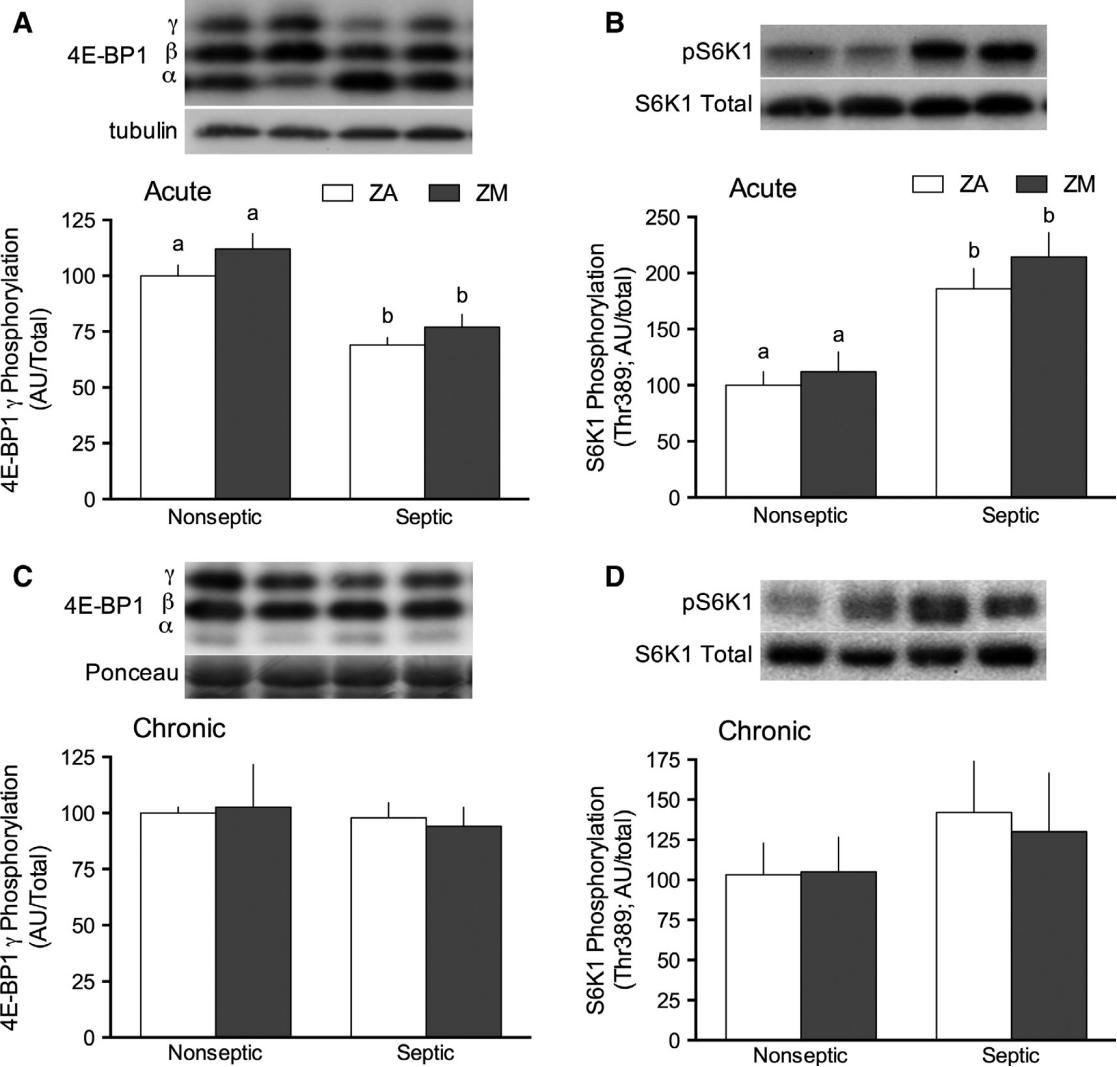
Effect of ZM and sepsis on phosphorylation of 4E‐BP1 and S6K1 in gastrocnemius. Representative western blots are above the respective bar graphs; specified bands were quantified and normalized to the ZA‐nonseptic group, set at 100 arbitrary units (AU). Total protein presented in each panel did not differ between groups (data not shown). (A) The *γ*‐isoform of 4E‐BP1, or highly phosphorylated form, is presented relative to total 4E‐BP1 in acute sepsis. The effect of sepsis (*P *< 0.0001) and diet (*P *= 0.02) were significant using a two‐way analysis of variance (ANOVA). (B) Phosphorylation of S6K1 relative to total S6K1 in acute sepsis. Effect of sepsis (*P *< 0.001) was significant by two‐way ANOVA; effect of diet or interaction was not significant. In chronic sepsis, 4E‐BP1 (C) and S6K1 (D) phosphorylation were unchanged with chronic sepsis or diet. Values with different letters are significantly different (*P *< 0.05). Values presented as mean ± SEM;* n *= 7–14 mice/group.

As mTOR is a central driver of the sepsis‐induced decrease in protein synthesis, its kinase activity was assessed by quantifying the phosphorylation of its downstream substrates, 4E‐BP1 and S6K1. Three bands were detected on the western blot of 4E‐BP1: the *α*‐, *β*‐ and *γ*‐isoforms correlating to increasing phosphorylation of the protein, respectively. The *γ*‐, or hyper‐phosphorylated isoform, was decreased by 40% in gastrocnemius during acute sepsis (Fig. 2A). Two‐way ANOVA analysis indicated an effect of the marginal dietary zinc restriction on 4E‐BP1 phosphorylation, although this effect did not show a significant difference between groups in post hoc testing. In contrast to the sepsis‐induced decrease in 4E‐BP1 phosphorylation and protein synthesis, acute sepsis lead to an increase in S6K1 phosphorylation in gastrocnemius, and this increase was comparable in ZA or ZM‐septic mice (Fig. 2B). In contrast to acute sepsis, the phosphorylation of 4E‐BP1 (Fig. 2C) and S6K1 (Fig. 2D) did not differ between groups regardless of sepsis or Zn concentration of the diet.

### Protein degradation

**Figure 3:**
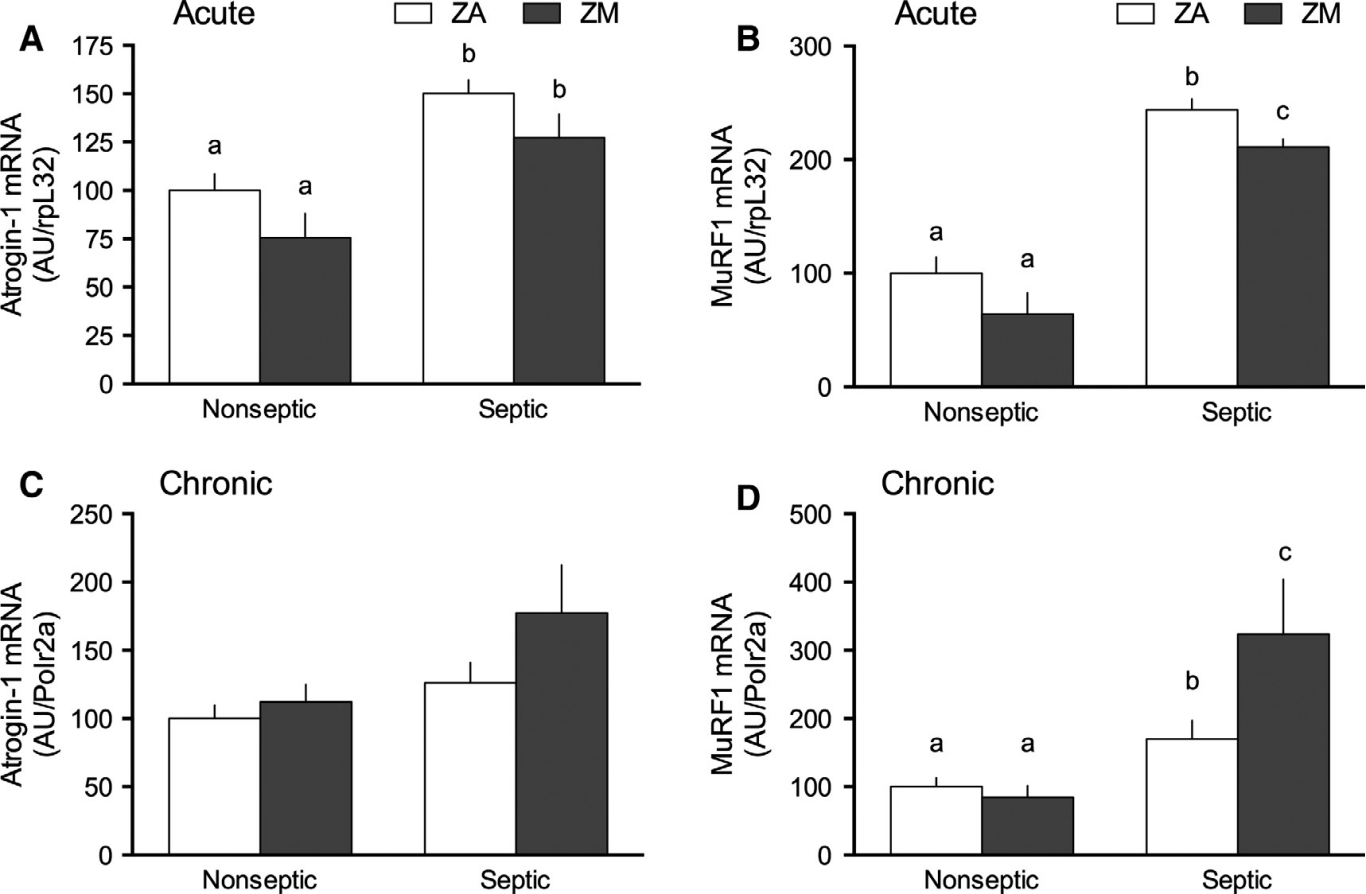
Effect of ZM on sepsis‐induced increase in atrogene mRNA content in gastrocnemius. Atrogin‐1 and MuRF1 mRNA relative to constituent levels of rpL32 in acute sepsis and Polr2a in chronic sepsis are presented with ZA‐nonseptic group values set at 100 AU. (A) In acute sepsis, atrogin‐1 had a sepsis (*P *< 0.0001) and ZM (*P *= 0.03) effect, but no interaction via two‐way analysis of variance (ANOVA). (B) In acute sepsis, MuRF1 also had a sepsis (*P *< 0.0001) and ZM (*P *= 0.01) effect, but no interaction via two‐way ANOVA. (C) In chronic sepsis, a sepsis effect (*P *= 0.02) but no ZM or interaction effects were found in atrogin‐1 mRNA. (D) In chronic sepsis, a sepsis (*P *= 0.0002) and interaction (*P *= 0.03) but no ZM effect were seen in two‐way ANOVA. Values with different letters are significantly different (*P *< 0.05). Values are presented as mean ± SEM;* n *= 8–11 mice/group.

The muscle‐specific E3 ubiquitin (Ub)‐ligases, MuRF1 and atrogin‐1, are surrogate markers for the Ub proteasome pathway (UPP) activity (Bodine et al. 2001). The mRNA content for both atrogin‐1 (Fig. 3A) and MuRF1 (Fig. 3B) was increased in gastrocnemius by acute sepsis. Acutely, the ZM diet had an effect on the mRNA content of atrogin‐1 and MuRF1 via two‐way ANOVA. Although atrogin‐1 mRNA did not differ between ZA and ZM mice, the sepsis‐induced elevation in MuRF1 was blunted by dietary Zn restriction compared to mice receiving adequate Zn diets (Fig. 3B). In chronic sepsis, there were no differences in post hoc analysis between the four groups for atrogin‐1 mRNA content (Fig. 3C). In contrast, chronic sepsis amplified the sepsis‐induced increase in MuRF1 mRNA (+325%) in ZM mice, compared to values from ZA‐nonseptic mice (Fig. 3D). Finally, the mRNA content for both ligases did not differ under nonseptic conditions between ZA and ZM mice in acute or chronic sepsis (Fig. 3A–D).

**Figure 4:**
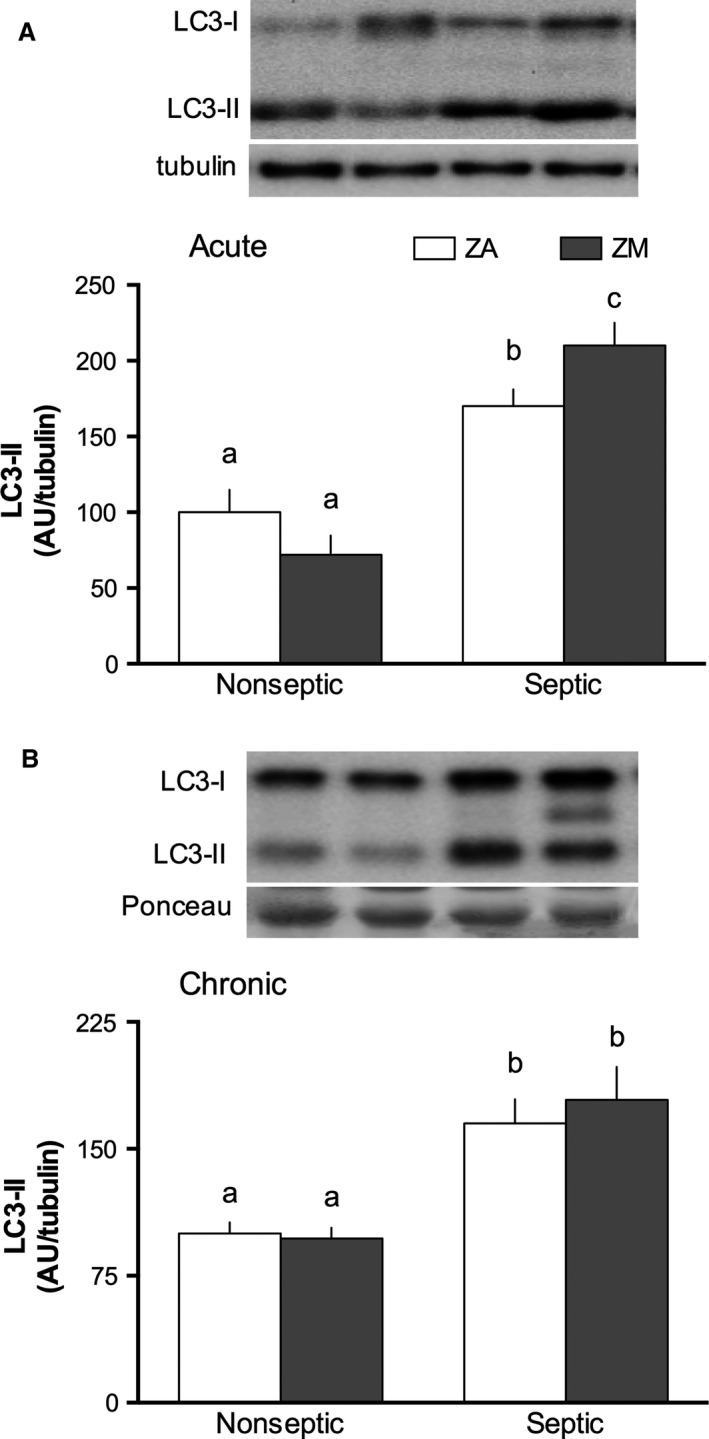
Effect of ZM on sepsis‐induced increases in LC3‐II. Quantification of LC3A/B‐II protein in the gastrocnemius of ZA and ZM groups in acute (A) and chronic (B) sepsis are presented with the ZA‐nonseptic group arbitrarily set to 100 AU. Representative western blots are presented above respective bar graphs. (A) Acute sepsis has a significant effect on LC3‐II via two‐way analysis of variance (ANOVA) (*P *< 0.001), and there was an interaction (*P *= 0.02) but no ZM effect was seen. (B) Chronic sepsis had an effect (*P *< 0.001), but no ZM effect or interaction were found in chronic sepsis. Values with different letters are significantly different (*P *< 0.05). Values presented as mean ± SEM;* n *= 8–11 mice/group.

LC3‐II, a membrane‐bound protein associated with autophagosomes, is an indicator of autophagy (Kabeya et al. 2000). LC3‐II content was increased in muscle in both the acute and chronic septic conditions (90 and 60%, respectively) in the ZA mice; a comparable sepsis‐induced increase in LC3‐II was detected in ZM mice (Fig. 4A & B).

### Muscle cytokine response

**Figure 5:**
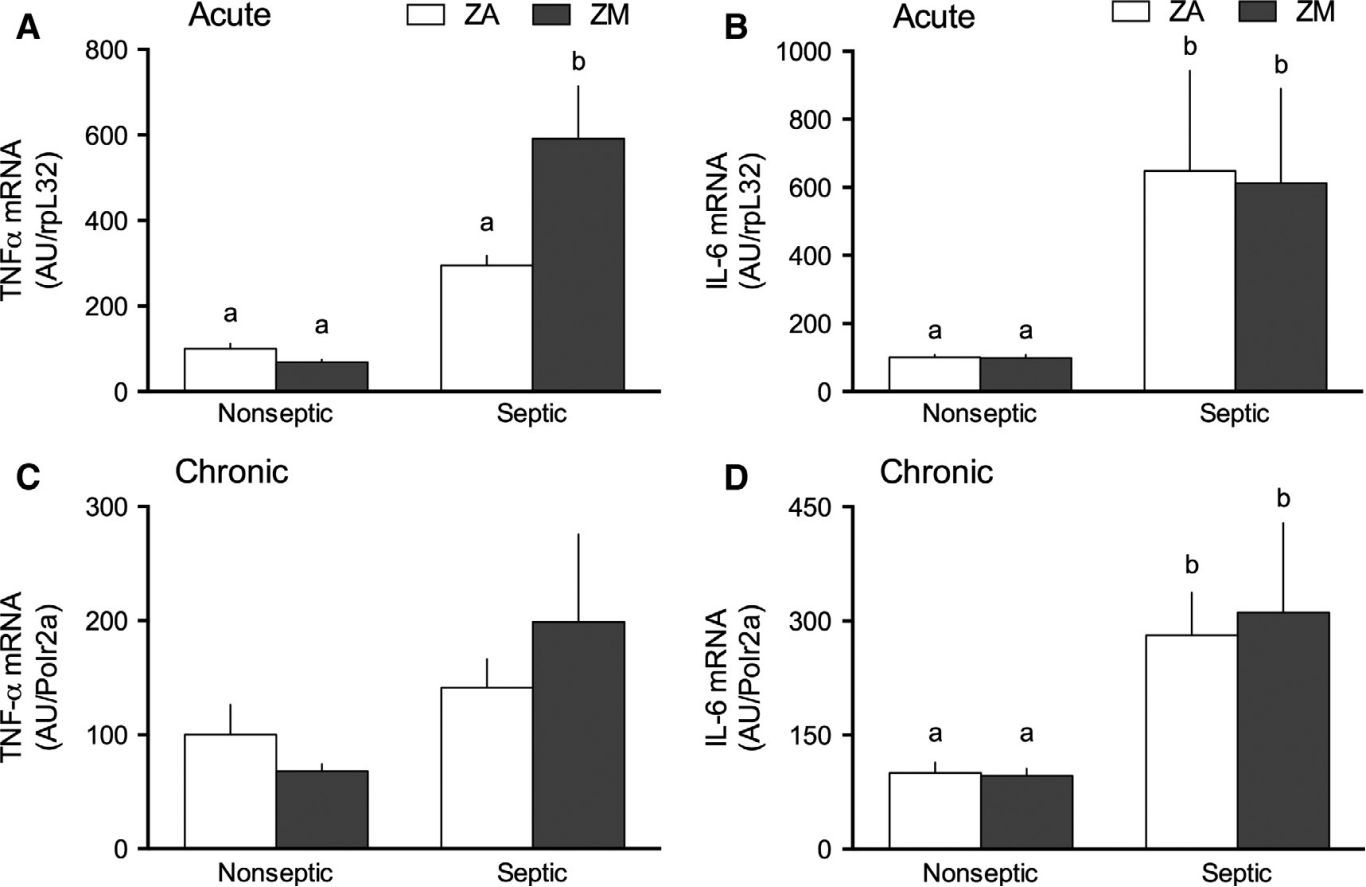
Effect of ZM and sepsis on cytokine mRNA content in gastrocnemius. Relative TNF‐*α* mRNA relative to constituent levels of rpL32 in acute sepsis (A) and Polr2a in chronic sepsis (C) are presented with ZA‐nonseptic group values set at 100 AU. Relative IL‐6 mRNA content with ZA‐nonseptic groups set at 100 AU. Statistical analysis on IL‐6 data were performed on the log of the values due to high variation in values in acute (*B*) and chronic (*D*) sepsis. *A*: In acute sepsis, a sepsis effect (*P *< 0.0001) and interaction (*P *= 0.02) were found in TNF‐*α* mRNA content. (B) In acute sepsis, sepsis was the only significant effect on IL‐6 mRNA content (*P *< 0.0001). (C) In chronic sepsis, no factor had a significant effect on TNF‐*α* mRNA content. (D) In chronic sepsis, sepsis had a significant effect on TNF‐*α* mRNA (*P *< 0.0001), but there were no significant diet or interaction effects. Values with different letters are significantly different (*P *< 0.05). All values are presented as mean ± SE;* n *= 8–11 for each group. Where absent, SEM bars are too small to visualize.

Skeletal muscle has the ability to mount an immune response to sepsis by stimulating the production of the cytokines including TNF‐*α* and IL‐6 (Frost et al. 2002). Acute sepsis increased TNF‐*α* mRNA content in the gastrocnemius almost threefold, compared to the nonseptic state in ZA mice, although this increase did not reach statistical significance. An interaction was seen with sepsis and dietary Zn, such that septic mice fed ZM had an exaggerated increase in TNF‐*α* mRNA content (greater than sevenfold), compared to ZA‐nonseptic mice (Fig. 5A). Acute sepsis also increased IL‐6 mRNA 650% in the gastrocnemius compared to the ZA‐nonseptic state, and this increase in IL‐6 was similar in the ZM mice (Fig. 5B).

In chronic sepsis, TNF‐*α* mRNA content did not differ between the four groups (Fig. 5C). In contrast, muscle IL‐6 mRNA remained persistently elevated above the nonseptic mice at day 5 (Fig. 5D). No difference in either cytokine was found between nonseptic or chronic septic mice fed the ZA or ZM diet (Fig. 5C & D).

## Discussion

This study assessed the effect of marginal dietary zinc deprivation and sepsis‐induced disturbances on the balance of muscle protein synthesis and degradation. Mild ZD, established by a 66% reduction in the dietary Zn intake, exacerbated the sepsis‐induced increase in skeletal muscle TNF‐*α* mRNA content at 24 h suggesting early amplification of local muscle‐specific inflammation responses in the septic state. Contrary to our hypothesis, however, a mild dietary restriction of Zn did not alter disturbances in protein synthesis, mTOR signaling, or protein degradation in the acute or chronic phases of sepsis. It is notable that measured endpoints related to protein balance were only studied in the gastrocnemius, a muscle with a relatively high predominance of fast‐twitch fibers. In our laboratory and others (Vary and Kimball 1992), the gastrocnemius appears to demonstrate a more consistent sepsis‐induced decrease in protein synthesis than soleus, although proteolytic activity may be higher in the soleus than in fast‐twitch muscles (Kadlcikova et al. 2004). Hence, we cannot exclude the possibility that mild ZD differentially affects slow‐twitch muscles.

In the nonseptic state, ZM did not alter the rate of protein synthesis or degradation (i.e., no change in atrogene mRNA or LC3B‐II protein). These data of marginal zinc deficiency are in contrast to previous studies investigating severe zinc deficiency, produced by feeding diets with less than 1 mg Zn/kg diet to rats, which reported decreases in the total protein content and rate of protein synthesis in skeletal muscle, as well as an increase in protein degradation (Giugliano and Millward 1984, 1987; Dørup and Clausen 1991). In this study, mild ZD under basal conditions did not significantly alter the protein balance in skeletal muscle supporting prior studies showing that in the nonstressed state, the ZM phenotype is seemingly normal (McCormick et al. 2015; Dempsey et al. 2012; Croxford et al. 2011).

Several studies have demonstrated that sepsis acutely decreases skeletal muscle protein synthesis and persistently suppresses protein synthesis in the chronic phase (Vary and Kimball 1992; Lang and Frost 2006; Vary et al. 1988; Jurasinski et al. 1995; Breuille et al. 1999). Consistent with these studies, our data show a 30% reduction in the rate of muscle protein synthesis, acutely following sepsis. In contrast, the reduction of protein synthesis did not persist at 5 days in our study, and there was even a trend toward increased protein synthesis in chronic sepsis, suggesting the start of a compensatory response to restore muscle protein content. One potential reason for these differences may be that a smaller gauge needle was used in our study to produce a milder sepsis (as death was not intended to be an endpoint). Thus, restoration of protein synthesis in chronic sepsis suggests an earlier resolution of the suppressed protein synthesis and earlier recovery. Additionally, the phosphorylation of 4E‐BP1 followed the trend of sepsis‐induced changes in protein synthesis in both acute and chronic sepsis, consistent with mTOR as the primary modulator of sepsis‐induced decrease in protein synthesis. Unlike 4E‐BP1 phosphorylation, the failure of sepsis to concurrently decrease S6K1 phosphorylation at 24 h (Lang et al. 2007; Lang and Frost 2004) was unexpected and the reason for this difference is not known and could suggest mTOR‐independent regulation of S6K1.

Similarly, the sepsis‐induced increase in the Ub proteasome system, evidenced by an elevation of the muscle‐specific E3 Ub‐ligating enzymes, atrogin‐1 and MuRF1, suggests an increase in proteolytic activity by the UPP during the acute phase of sepsis. In the chronic phase, there is a mild elevation of MuRF1 but atrogin‐1 does not concurrently increase with sepsis, thus it is unlikely that the overall activity of the UPP is increased in chronic sepsis. Previous work reported elevated mRNA for both enzymes acutely after sepsis; however, atrogin‐1 was normalized by day 3 while MuRF1 remained elevated (Frost et al. 2007). Again, differences between our data and previous reports in the chronic phase of sepsis may be explained by a less severe septic insult leading to either a lower initial expression or a faster resolution of sepsis‐induced factors inducing these E3 Ub‐ligases.

Muscle autophagy has been observed to be elevated in both acute and chronic phases of sepsis. Two mechanisms regulate autophagy to various extents: the Akt/forkhead box O3 (FoxO3) signaling and the phosphorylation of Unc‐51‐like autophagy‐activating kinase 1 (ULK1) protein by mTOR (Jung et al. 2009; Zhao et al. 2007). Our data corroborate prior studies (Steiner et al. 2015), in which a sepsis‐induced increase in skeletal muscle autophagy is evidenced by an elevation of LC3‐II protein at 24 h and is associated with a downregulation of mTOR signaling. Furthermore, 5 days after CLP, LC3‐II remained elevated despite restoration of mTOR signaling. Our data suggest that in this more mild form of sepsis, despite normalization of protein synthesis and degradation via the UPP, autophagy is persistently elevated. Continued protein degradation has been previously reported to occur via other pathways in chronic sepsis (Voisin et al. 1996), but autophagy in chronic sepsis has not been well studied and may play a role in delayed recovery of muscle protein.

Critical illness and sepsis are characterized by an elevation of systemic cytokines; additionally, organs such as skeletal muscle also increase synthesis of cytokines, TNF‐*α* and IL‐6, in response to endotoxin from gram‐negative bacteria (Frost et al. 2002). TNF‐*α* can induce both global muscle wasting (Moldawer and Copeland 1997) as well as act in a paracrine or autocrine fashion to inhibit muscle protein synthesis (Frost et al. 1997). IL‐6 may also promote muscle wasting by increasing protein breakdown and decreasing synthesis (Fujita et al. 1996; Tsujinaka et al. 1996). Sepsis acutely elevated both TNF‐*α* and IL‐6 mRNA in the gastrocnemius, and as a result, these local cytokines may participate in the reduction of protein synthesis and increase in protein degradation at this early time point. At 5 days, however, the sepsis‐induced increase in TNF‐*α* mRNA normalized in a temporally similar manner to protein synthesis and the UPP; however, IL‐6 production in muscle remained elevated. It is difficult to speculate the impact of increased IL‐6, but it may play a role in the concomitant increase in autophagy as seen at 5 days post‐CLP.

In prior reports (Knoell et al. 2009), severe ZD in the murine model resulted in an amplified inflammatory response to acute sepsis. Marginal dietary Zn deprivation in this study also enhanced the sepsis‐induced increase in TNF‐*α* mRNA content in the muscle, but only in the acute phase. However, this exaggerated increase in the locally produced cytokine did not impact the sepsis‐induced decrease in muscle protein synthesis, mTOR signaling, or autophagy. In ZM mice, the sepsis‐induced changes in MuRF1 were time‐dependent. That is, the sepsis‐induced elevation of MuRF1 was blunted in the acute phase but was exaggerated in chronic sepsis. While it seems unlikely that the continued elevation of MuRF1 alone in ZM‐septic mice leads to a global increase in proteolysis, it is possible that the increase in E3 ligase activity promotes the selective breakdown of one or more muscle proteins not yet identified.

In conclusion, to the best of our knowledge, these are the first studies to explore the response to abdominal sepsis in a model of mild diet‐induced zinc deficiency. This study used the CLP model of peritonitis, at both an early and later time point in the progression of sepsis, which is generally considered to be more clinically relevant than bolus injections of endotoxin or live bacteria (Iskander et al. 2013). While significant or severe deficiencies may be expected to produce aberrant responses, our studies suggest that milder forms of zinc deficiency may provide a more detailed understanding of the more commonly observed deficiency states in the Western countries. Our data confirm prior observations that in murine models of sepsis, decreased muscle protein synthesis is associated with a decrease in mTOR signaling as well as increased muscle protein degradation. Against a background of mild dietary zinc deprivation, alterations were observed in select aspects of muscle cytokine response and expression of the Ub proteasome pathway. Initially after sepsis, our data suggest that mild deficiencies do not alter the capability of the body to support the acute responses and that zinc supplementation may not be required during the early phase of illness. It is important to note that our observations do not exclude the possibility that long‐term consequences of mild zinc deficiencies may be observed in other metabolically and immunologically active organs, resulting in disturbances in healing and recovery. Thus, further studies are required to better understand the effects of marginal Zn intake on subtle or delayed consequences during long‐term convalescence from acute illness.

## Conflict of Interest

We have no conflicts or financial disclosures to report in relation to this work.
